# Retraction: The resistant effect of SIRT1 in oxidative stress-induced senescence of rat nucleus pulposus cell is regulated by Akt-FoxO1 pathway

**DOI:** 10.1042/BSR20190112_RET

**Published:** 2026-09-09

**Authors:** Junsheng He, Ailiang Zhang, Zhiwen Song, Shiwu Guo, Yuwei Chen, Zhiyuan Liu, Jinlong Zhang, Xu Xu, Jinbo Liu, Lei Chu

**Affiliations:** 1 Department of Spinal surgery, The Third Affiliated Hospital of Soochow University, Changzhou 213003, China; 2 Department of Orthopedics, The Affiliated Wujin Hospital of Jiangsu University, Changzhou 213003, China; 3 Department of Orthopedics, The Second Affiliated Hospital of Chongqing Medical University, Chongqing 400010, China

**Keywords:** FoxO1, IDD, oxidative stress, PI3K/Akt, Senescence, sirtuins

This article is being retracted from *Bioscience Reports* at the request of the Editor-in-Chief and the Editorial Board.

The authors contacted the journal following reader comments on Pubpeer to alert the Editorial Board to concerns within the article. These included high similarities between the Figure 5F SRT1720 + H2O2 panel and the Figure 8I RSV + H2O2 panel, between the Figure 6A Akt and B-actin western blot bands, as well as concerns regarding the choice of antibody used in the experiment.

The authors fully cooperated and partially provided original data requested along with explanations, which included errors during figure assembly. However, some raw data for Figure 8 were missing, and some alterations and transformations identified. The authors also agreed that the antibody listed in the methods is incorrect: a mistake that resulted from vendor search engine nomenclature errors and ambiguous product tagging.

Since multiple conclusions are impacted by the problematic data and errors, it is deemed most appropriate by the Editor-in-Chief and the Editorial Board to retract the article.

The authors do not agree to the retraction.

